# Efficacy of Cerebral Autoregulation in Early Ischemic Stroke Predicts Smaller Infarcts and Better Outcome

**DOI:** 10.3389/fneur.2017.00113

**Published:** 2017-03-24

**Authors:** Pedro Castro, Jorge Manuel Serrador, Isabel Rocha, Farzaneh Sorond, Elsa Azevedo

**Affiliations:** ^1^Department of Neurology, São João Hospital Center, Faculty of Medicine, University of Porto, Porto, Portugal; ^2^Department of Pharmacology, Physiology and Neuroscience, Rutgers Biomedical Health Sciences, Newark, NJ, USA; ^3^Department of Veteran Affairs, Veterans Biomedical Institute, War Related Illness and Injury Study Center, East Orange, NJ, USA; ^4^Cardiovascular Electronics, National University of Ireland Galway, Galway, Ireland; ^5^Faculty of Medicine, Cardiovascular Centre, Institute of Physiology, University of Lisbon, Lisbon, Portugal; ^6^Department of Neurology, Division of Stroke and Neurocritical Care, Northwestern University Feinberg School of Medicine, Chicago, IL, USA

**Keywords:** cerebral autoregulation, blood pressure, stroke, ischemic stroke, transcranial Doppler

## Abstract

**Background and purpose:**

Effective cerebral autoregulation (CA) may protect the vulnerable ischemic penumbra from blood pressure fluctuations and minimize neurological injury. We aimed to measure dynamic CA within 6 h of ischemic stroke (IS) symptoms onset and to evaluate the relationship between CA, stroke volume, and neurological outcome.

**Methods:**

We enrolled 30 patients with acute middle cerebral artery IS. Within 6 h of IS, we measured for 10 min arterial blood pressure (Finometer), cerebral blood flow velocity (transcranial Doppler), and end-tidal-CO_2_. Transfer function analysis (coherence, phase, and gain) assessed dynamic CA, and receiver-operating curves calculated relevant cut-off values. National Institute of Health Stroke Scale was measured at baseline. Computed tomography at 24 h evaluated infarct volume. Modified Rankin Scale (MRS) at 3 months evaluated the outcome.

**Results:**

The odds of being independent at 3 months (MRS 0–2) was 14-fold higher when 6 h CA was intact (Phase > 37°) (adjusted OR = 14.0 (IC 95% 1.7–74.0), *p* = 0.013). Similarly, infarct volume was significantly smaller with intact CA [median (range) 1.1 (0.2–7.0) vs 13.1 (1.3–110.5) ml, *p* = 0.002].

**Conclusion:**

In this pilot study, early effective CA was associated with better neurological outcome in patients with IS. Dynamic CA may carry significant prognostic implications.

## Introduction

Reperfusion and neuroprotection are the current mainstays of acute ischemic stroke (IS) management. In this regard, arterial blood pressure (ABP) management may play a central role to maintain optimal perfusion within the vulnerable ischemic penumbra (1, 2). Unfortunately, several clinical trials in ABP modulation had no effect on prognosis (2) and, therefore, the corresponding current guidelines remain evasive (3). Perhaps, the crucial factor is not ABP *per se*, but rather how cerebral blood flow can adapt to pressure changes and/or demand, i.e., cerebral autoregulation (CA) (4).

Dynamic CA (dCA) can be assessed using transfer function analysis (TFA) between spontaneous oscillations of ABP and cerebral blood flow velocity (CBFV) (4). CA has been studied in acute stroke (5) with conflicting results (5–8), but the early hours, where penumbra is more vulnerable, has been largely ignored.

Therefore, we aimed to assess dCA within 6 h of IS symptoms and its relationship with final infarct volume and 90-day functional outcome.

## Materials and Methods

### Study Population

São João Hospital center ethical committee approved the study. Written informed consent was obtained. We included consecutive patients with middle cerebral artery (MCA) territory acute IS, admitted to our stroke unit. Ultrasound studies (Vivid *e*; GE) excluded hemodynamically significant extra- or intracranial stenoses. Patients with MCA proximal occlusion were excluded, as it prevented monitoring.

### Monitoring and Data Analysis

Evaluations were carried out at stroke unit in supine position. We monitored for 10 min CBFV with transcranial Doppler M1-MCA (BoxX-DWL, Germany), ABP with Finometer MIDI (FMS, Netherlands), heart-rate and end-tidal carbon dioxide (EtCO_2_) with capnograph (Respsense/Nonin, Netherlands). Systolic, diastolic, and mean values of ABP (MBP) and of CBFV (MFV) were calculated (4). TFA assessed dCA by calculating coherence, gain, and phase parameters from beat-to-beat MFV and MBP spontaneous oscillations in low frequency range (0.03–0.15 Hz) (4) as previously detailed (9).

### Outcomes and Statistics

Baseline National Institutes of Health Stroke Scale (NIHSS) scores were calculated. Independence, modified Rankin Scale (0–2), at 90 days determined the outcome by a stroke physician blinded for the initial assessment. Head CT (Siemens Somaton/Emotion Duo, Germany) at 24 h measured infarct volume with ABC/2 formula.

Shapiro–Wilk test determined normality. Mann–Whitney and χ^2^ tests compared hemodynamic measurements between subgroups. ROC analysis found relevant cutoff values. After dichotomization, multivariate logistic regression calculated the odds ratio. Relationship between continuous variables was determined by Spearman’s correlation and adjusted with multivariate linear regression models. Level of significance was *p* < 0.05.

## Results

**Figure 1:**
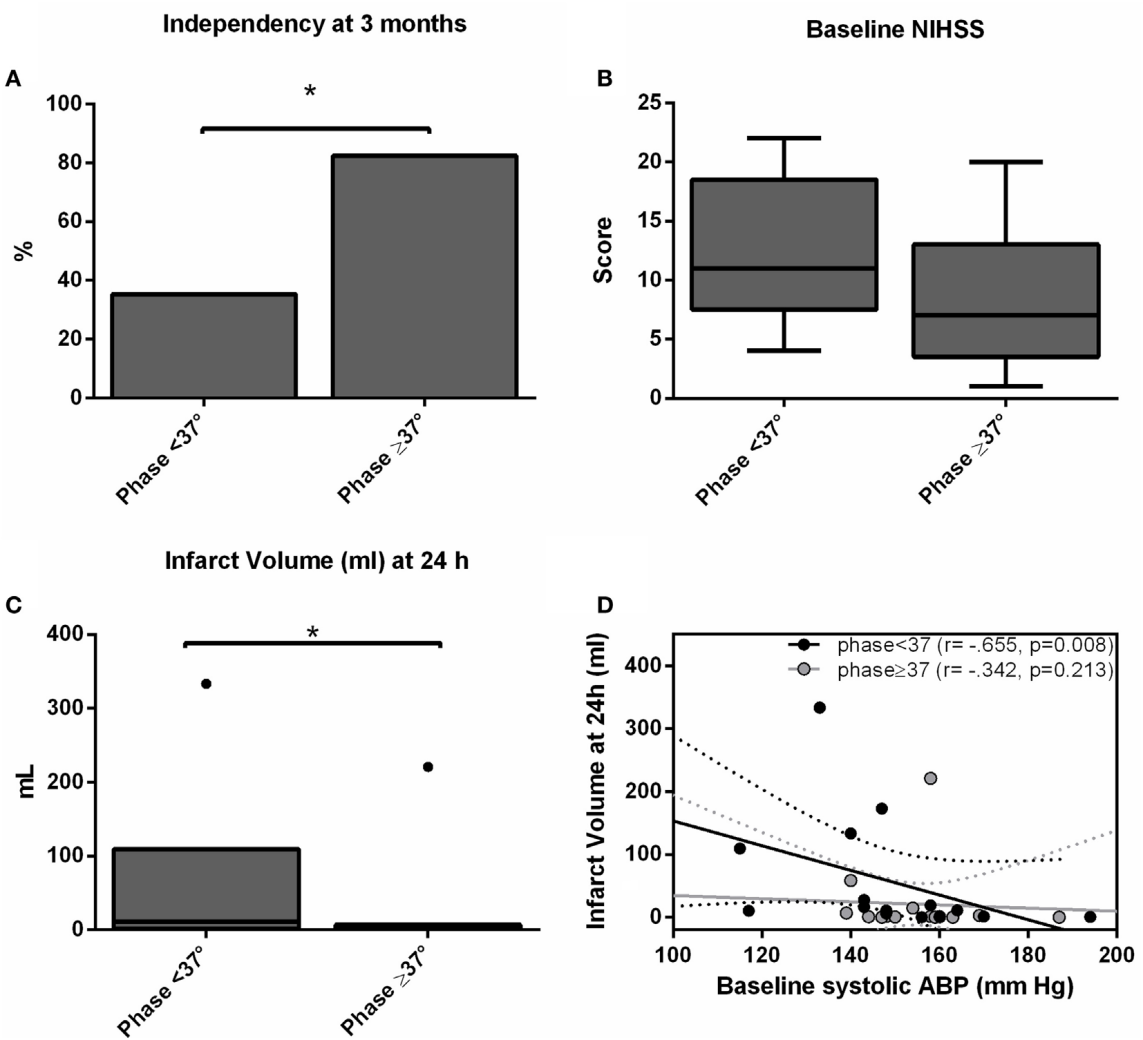
**Independency at 3 months (A), baseline National Institutes of Health Stroke Scale score (B) and infarct volume (C) accordingly subgroups of worse (phase < 37°) and better (phase ≥ 37°) dynamic CA (dCA) on the ischemic hemisphere**. Bars represent % and box-and-whiskers plots medians/interquartile ranges. Significant results (*p* < 0.05) are topped by asterisks. **(D)** Scatter plot showing infarct volumes at 24 h (ml) plotted against baseline systolic arterial blood pressure level separated by subgroups of worse (phase <37°, black dots) and better (phase ≥37°, gray dots) dCA, and respective linear regression lines and 95% confidence interval.

We recruited 30 patients characterized in Table 1. The relationship between dCA and outcome is presented in Table 2. Independence at 3 months was associated with higher phase (*p* = 0.024) and lower gain (*p* = 0.045) in the stroke hemisphere within 6 h of onset. ROC curve analysis found best cutoffs, associated with independency, in phase at 37° (affected side, AUC = 0.713, *p* = 0.028; sensitivity 70%, specificity 79%) but gain underperformed (AUC = 0.654, *p* = 0.112). Based on these cutoffs, independency at 3 months (Figure 1A) could be predicted by phase level in the affected side [phase ≥ 37°, adjusted OR = 14.0 (IC 95% 1.7–74.0), *p* = 0.013] when adjusted to baseline NIHSS and age. Additionally, lower infarct volumes at 24 h (Figure 1C) were measured in subgroups of higher phase in the affected side [median (range) 1.1 (0.2–7.0) vs 13.1 (1.3–110.5) ml, *p* = 0.002]. Low and high phase subgroups were also not significantly different in baseline NIHSS (Figure 1B, *p* = 0.062). When phase was analyzed as a continuous variable, it correlated with stroke volume in the affected side (*r* = −0.444, *p* = 0.020) but not contralateral (*r* = −0.125, *p* = 0.409). In multivariate linear regression, only NIHSS significantly predicted infarct volume at 24 h (*p* = 0.002) but not phase (*p* = 0.457). Baseline systolic ABP was inversely correlated with infarct volume at 24 h (*r* = −0.665, *p* = 0.008) but only in the subgroup with lower phase in the infarct side (Figure 1D).

**Table 1:** **Patients characteristics according to outcome at 3 months**.

	Total	Independency	
		Yes	No
		
	*N* = 30	*N* = 17	*N* = 13
Gender male, *n* (%)	16 (53)	9 (53)	5 (39)
Age, years (mean ± SD)	69 ± 13	63 ± 13	76 ± 9*
BMI, kg m^−2^ (mean ± SD)	26.9 ± 5.5	26.9 ± 4.0	26.9 ± 7.0
Atrial fibrillation, *n* (%)	11 (37)	4 (23)	7 (55)
Hypertension, *n* (%)	20 (67)	9 (53)	11 (85)
Diabetes mellitus, *n* (%)	12 (40)	4 (25)	8 (60)
Dyslipidemia, *n* (%)	22 (74)	12 (71)	10 (76)
Tobacco, *n* (%)	5 (17)	3 (18)	2 (15)
Large vessel atherosclerosis	4 (13)	2 (10)	2 (20)
Cardioembolic	11 (37)	4 (20)	7 (60)
Small vessel disease, *n* (%)	3 (10)	1 (1)	2 (5)
Undetermined, *n* (%)	8 (27)	2 (10)	6 (55)
Thrombolysis, *n* (%)	20 (67)	11 (55)	9 (53)
Baseline NIHSS, median (IQR)	9 (5–15)	6 (4–13)	12 (8–20)*

*BMI, body-mass index; NIHSS, National Institutes of Health Stroke Scale*.

*P < 0.05 significance value of Mann–Whitney. Values in mean ± SD.

**Table 2:** **Cerebral autoregulation and outcome at 3 months**.

	Total	Independency	
		Yes	No
		
	*N* = 30	*N* = 17	*N* = 13
Heart rate, bpm	70 ± 11	70 ± 10	71 ± 12
Systolic ABP, mmHg	136 ± 23	134 ± 20	138 ± 23
Mean ABP, mmHg	81 ± 14	84 ± 15	77 ± 17
Diastolic ABP, mmHg	54 ± 13	57 ± 13	50 ± 17
EtCO_2_, mmHg	37 ± 6	36 ± 5	37 ± 5
*Cerebral hemodynamics*			
*Infarct hemisphere*			
MFV, cm/s	42 ± 15	46 ± 18	49 ± 13
Coherence, a.u.	0.5 ± 0.2	0.5 ± 0.2	0.5 ± 0.2
Gain,%/mmHg	1.0 ± 0.4	0.8 ± 0.2	*1.1 ± 0.5
Phase, degrees	36 ± 38	50 ± 25	*21 ± 47
*Non-infarct hemisphere*			
MFV, cm/s	50 ± 16	50 ± 17	46 ± 12
Coherence, a.u.	0.5 ± 0.2	0.5 ± 0.2	0.6 ± 0.2
Gain,%/mmHg	1.1 ± 0.6	0.9 ± 0.2	1.1 ± 0.6
Phase, degrees	43 ± 33	48 ± 21	39 ± 40

*Interhemispheric = ipsilateral-minus-contralateral values; MFV, mean flow velocity; ABP, arterial blood pressure; EtCO_2_, end-tidal carbon dioxide; a.u., arbitrary units*.

*P < 0.05 significance value of Mann–Whitney. Values in mean ± SD.

## Discussion

We showed that the efficacy of dCA during the first 6 h after symptom onset is associated with smaller infarct volumes at 24 h and better neurological outcome at 3 months.

Transfer function analysis of the spontaneous ABP and CBFV oscillations is increasingly used to assess dCA in a number of neurovascular disorders (7–10). The phase of this relationship, which represents the time delay between these oscillating waveforms, has emerged as a significant predictor of outcome. Lower phase shift (ineffective CA) has been linked to carotids or MCA stenosis (11) or development of vasospasm after subarachnoid hemorrhage (10). In patients with IS, phase has also been linked to stroke severity (5, 7). The impaired CA can be also related to patient medical conditions not addressed in this study. For example, impaired cerebral autoregulation in patients with sleep apnea has been linked to an increased risk of stroke (12). Our findings, which build on these prior studies, show that effective dCA, as demonstrated by higher phase shift, is linked to smaller stroke volumes and better neurological outcome. Moreover, consistent with prior work where a phase >30 represents effective or intact autoregulation (4, 5, 9), we also found a cutoff value of 37° for phase that was predictive of neurological independence at 3 months and smaller stroke volumes at 24 h.

Interestingly, we also found that a lower systolic ABP is associated with larger infarcts but only if CA is impaired in the infarct side (phase <37°). This observation enhances the biological plausibility of the link between phase (dCA), stroke volume and clinical outcome, since lower ABP would only endanger the ischemic penumbra with further hypoperfusion if CA was impaired. Taken together, CA assessment could, therefore, identify patients who would benefit from BP augmentation in future clinical trials (13) Perfusion imaging, instead of CA assessment, may have been more helpful to explain larger infarcts at 24 h by estimation of initial penumbra area. However, an impaired CA at baseline could itself be responsible for this larger penumbra. The question remains to be answered in future studies with correlative measurements with perfusion scanning.

In line with prior studies (5), gain seems not to be a good marker for stroke outcome. Nevertheless, lower gain values (more effective CA) on the stroke side seemed to be associated with independence at 3 months.

This study has some limitations. As it is a pilot study, we enrolled a small number of subjects. Regarding the TCD method, there are limitations inherent to CA assessment with TCD (4), as some non-stationary conditions (e.g., agitation, mental changes) might turn linear methods like TFA less reliable. Also, M1 occlusions could not be assessed. As CA was assessed after IV thrombolysis within 6 h of symptoms, non-occluded M1 cases in this study include recanalyzed MCA or branch occlusions while those who were excluded due to M1 occlusion are mostly non-recanalized MCA. Having said that, we still can see this as a limitation but occluded M1 after IV thrombolysis is itself a maker for very bad prognosis and we believed that CA assessment would not add any significant contribution in this scenario; we also monitored this excluded cases and only 1/16 (6%) was independent at 3 months and all had total MCA area involvement. So, what our study points out is that even if we recanalyze the MCA artery <6h, those with better CA (phase ≥37°) will have higher chance of being independent at 3 months.

Concerning the infarct volume, we used CT scan, which is not as reliable as MRI. However, most of the stroke patients had easily identifiable partial or total areas of MCA infarct. Although CT scan is a coarse measure, we believe that the overall results were not influenced by this method.

1. In summary, we showed that the efficacy of dCA in the early hours of IS is linked to infarct volume at 24 h and neurological outcome at 3 months. Rapid bedside assessment of CA may help to identify a high risk population with impaired CA who would benefit from different BP management.

## Ethics Statement

This study was carried out in accordance with the recommendations of São João Hospital center ethical committee with written informed consent from all subjects. All subjects gave written informed consent in accordance with the Declaration of Helsinki. The protocol was approved by the São João Hospital center ethical committee.

## Author Contributions

PC reviewed the literature, designed the study, extracted the data, analyzed the results, and wrote the paper. EA designed the study, analyzed the results, and co-wrote the paper. IR and JS designed the study, analyzed the results, and reviewed the paper. FS reviewed the literature, designed the study, analyzed the results, and co-wrote the paper.

## Conflict of Interest Statement

The authors declare that the research was conducted in the absence of any commercial or financial relationships that could be construed as a potential conflict of interest.
